# Concept mapping to reach consensus on a 6‐month exclusive breastfeeding strategy model to improve the rate in Northeast Thailand

**DOI:** 10.1111/mcn.12823

**Published:** 2019-05-13

**Authors:** Thiwawan Thepha, Debbie Marais, Jacqueline Bell, Somjit Muangpin

**Affiliations:** ^1^ Department of Advanced Midwifery, Faculty of Nursing Khon Kaen University Khon Kaen Thailand; ^2^ Warwick Medical School University of Warwick Coventry UK; ^3^ College of Life Sciences and Medicine University of Aberdeen Aberdeen UK

**Keywords:** concept mapping, intervention, North‐east Thailand, policy, 6‐month exclusive breastfeeding

## Abstract

**Background**

In implementation research, it is essential to involve all stakeholders in the development of complex interventions to ensure that the proposed intervention strategy is relevant and acceptable to the target area and group. The aim of this study was to involve stakeholders in conceptualising, developing, and prioritising a feasible intervention strategy to improve the 6‐month exclusive breastfeeding rate in North‐east Thailand. Concept mapping was used in a purposive sample including health care volunteers, health care professionals, and community leaders. During the first meeting, stakeholders (*n* = 22) expressed the generation of feasible interventions. During the second meeting, participants (*n* = 21) were asked to individually rate the feasibility of each intervention and to group them into relevant categories to enable multidimensional scaling and hierarchical cluster analysis. The outputs of analysis included the intervention list, cluster list, point map, point rating map, cluster map, and cluster rating map. All of these were shared with stakeholders (*n* = 17) during the third meeting to reach consensus on an intervention model. The final proposed intervention strategy included 15 feasible interventions in five clusters: health care services, community services, and education packages for parents, family members, and communities. These interventions were prioritised for implementation over a 3‐year period. Once the feasibility of each intervention is established, the proposed model could be implemented and incorporated into local health policy. After assessing intervention effectiveness, each intervention could be scaled up to other middle‐income countries to help improve overall maternal and child survival.

**Key messages**

- A feasible 3‐year intervention strategy model was developed to increase the 6‐month exclusive breastfeeding rate and improve the impact of breastfeeding on maternal and child survival, specifically in North‐east Thailand.
- This strategy included 15 feasible interventions grouped in five clusters; health care services, community services, and education packages for parents, family members, and communities.
- The use of concept mapping enabled consensus building, and the outputs can be replicated and scaled up in other middle‐income countries.

## 1 INTRODUCTION

In order to support maternal and child survival, exclusive breastfeeding (EBF) is currently recommended for the first 6 months of an infant's life (WHO, 2001). In Thailand, the 6‐month EBF rate was reported as only 15% in 2009 (National Statistical Office Thailand, 2010) and worryingly dropped to 12% in 2013 (National Statistical Office Thailand, 2013). Regionally, the 6‐month EBF rates for the North‐east, South, North, and Central regions were 27%, 10%, 9%, and 6%, respectively, in 2009 (National Statistical Office Thailand, 2010). Although most regions saw increases in EBF rates in 2013, the rates in North‐east Thailand had reduced significantly to 14% (National Statistical Office Thailand, 2013). To tackle this reduction and promote the use of 6‐month EBF in North‐east Thailand, effective interventions must be developed.

Both governmental and nongovernmental organisations have previously implemented interventions to protect, promote, and support breastfeeding in Thailand. Example interventions include the Baby Friendly Hospital Initiative, introduction of designated breastfeeding areas in the workplace, provision of antenatal education packages and postnatal support as well as community projects, and interventions to increase the self‐efficacy of mothers and health care professionals (Budsaengdee, Kantaruksa, & Chareonsanti, 2013; Hangchaovanich & Voramongkol, 2006; Kupratakul, Taneepanichskul, Voramongkol, & Phupong, 2010; Prasopkittikun & Sangperm, 2017; Sinthukot & Jirapaet, 2014). Nevertheless, these interventions failed to effectively increase 6‐month EBF rates in North‐east Thailand. The attitudes and strategies adopted when implementing these interventions, and several contextual factors could explain the inefficiency of these interventions. Specifically, interventions were implemented using a top‐down approach without targeting specific regions; they lacked appropriate targets (short‐term/long‐term) and mechanisms for monitoring, whereas factors such as health inequalities; lack of support within communities, family, or the workplace; and postpartum mother migration may have also added to the poor effectiveness of these interventions (Apasakul, 2016; National Statistical Office Thailand, 2013). To address these possible reasons, contextualisation and sustainability of proposed interventions, including policies, must be considered.

Four main factors influence EBF globally: factors related to the mother (Bosi et al., 2015; Kim & Chapman, 2013; Kounnavong et al., 2013; UNICEF, 2018), the infant (Desai et al., 2014; Kermani, Nedaeifard, Tehrani, Nateghi, & Fazeli, 2012; Samuel, Thomas, Bhat, & Kurpad, 2012; Tan, 2011), the health care environment (Ahmad, Sughra, Kalsoom, Imran, & Hadi, 2012; Brown, Raynor, & Lee, 2011; Marks & O'Connor, 2014; Radzyminski & Callister, 2015), and the social environment (Hmone, Dibley, Li, & Alam, 2016; Hoddinott, Craig, Britten, & McInnes, 2012; Nduna, Marais, & Wyk, 2015). Although similar, when specifically identifying facilitators and barriers to 6‐month EBF in North‐east Thailand, there were some unique factors identified as either a facilitator, a barrier, or both (Thepha, Marais, Bell, & Muangpin, 2018). In addition to these factors, our previous study identified several barriers and/or facilitators specific to 6‐month EBF in North‐east Thailand (Thepha et al., 2018). Specifically, facilitators were breastfeeding knowledge, intention to breastfeed, family support, social media platforms (using, for example, Facebook, webpages or other communication applications, or other online forums to search for exchange EBF knowledge or offer or get advice/support), health care facilities, and health care professional knowledge, whereas perceptions, having another child, promotion of formula milk, stress, workplace context, government policy, and conflicting advice were reported as barriers. Mothers’ individual and traditional beliefs and hospital policy were reported as both facilitators and barriers (Thepha et al., 2018).

It is therefore clear that increasing the rate of 6‐month EBF in North‐east Thailand, which is a complex issue, is challenging because various facilitators and barriers need to be addressed. Complex issues cannot be addressed by only one intervention; therefore, a complex and multifaceted intervention strategy needs to be developed (Wolff, 2001; Craig et al., 2006). Developing a complex intervention includes four stages, namely, development, feasibility, evaluation, and implementation (Campbell et al., 2000; Craig et al., 2013; Dieppe, 2006), which relates well to the three steps in implementation research, which include initiation and scoping; and planning and design and implementation, iterative improvement, and scaling up (Society for implementation science in nutrition (SISN, 2017). Framing the research on existing evidence within the relevant context as well as utilising experiential knowledge by consulting stakeholders to set the agenda and identify possible solutions within constraints (Craig et al., 2013; Society for implementation science in nutrition (SISN, 2017) is essential given the complexity of implementation. Accordingly, this study aimed to develop a context‐sensitive and acceptable intervention model to promote 6‐month EBF in North‐east Thailand.

## 2 METHODS

### 2.1 Concept mapping

Concept mapping (CMP) has been used in many health and social sciences studies to inform health policy tapping into the group's ideas (Donnelly, Huff, Lindsey, McMahon, & Schumacher, 2005; Kupratakul et al., 2010; Rao et al., 2005). It is a systematic and collaborative group process to conceptualise the ideas of stakeholders (Kane & Trochim, 2007; Sutherland & Katz, 2005; Trochim, 1989; Jackson & Trochim, 2002). This process identifies the open contribution of participants or stakeholders' ideas on a specific issue as contextual based and tacit knowledge and organises relevant ideas to capture them in the form of picture or map, which is also known as “geography of thought.” It can easily capture and understand overall ideas (Kane & Trochim, 2007; Trochim, 1989; Rosas & Camphausen 2007; Rosas & Kane, 2012).

CMP uses both qualitative and quantitative approaches and follows six steps: preparation, generation of interventions, structuring of interventions, representation of interventions, interpretation of results, and utilisation of results (Kane & Trochim, 2007; Rosas, 2007; Rosas & Kane, 2012; Sutherland & Katz, 2005; Trochim, 1989). In the preparation step, the facilitator needs to select participants and set the desired outcomes addressing research objectives. In the generation of possible intervention steps, the facilitator prompts the participants to freely express their ideas. The interpretation and utilisation of results are two important steps that challenge the facilitator to present concept maps and identify outcomes that respond to the objectives of the research (Kane & Trochim, 2007; Sutherland & Katz, 2005; Trochim, 1989). This study received ethical approval from the University of Aberdeen (No. CERB/2015/3/1147) and the Khon Kaen hospital ethical committee, Thailand (No. KE 58059).

### 2.2 Participants

Participants include people within an organisation or persons who are knowledgeable, committed, and potentially involved in the research question of interest (Kane & Trochim, 2007), protecting and promoting breastfeeding in this case. Eligible participants included persons responsible for maternity services in the Khon Kaen district of the North‐east Thailand including community leaders, health professionals at the subdistrict level, community nurses, health volunteers representing family members, and nurses from private hospitals. Purposive sampling was used to recruit eligible stakeholders from each group to provide contextual and experiential knowledge. Both governmental and private health institutions within the Khonkaen district were contacted to identify potential participants. Additionally, health professionals from neighbouring regions in Thailand who had achieved success in increasing their own region's 6‐month EBF rates were also invited.

Although the recommended sample size for CMP studies vary (Kane & Trochim, 2007), 20 participants have been suggested being effective (Trochim, 1989). Ideally, all participants should attend every meeting although this is not essential, and the data can be used even though they did not join each meeting (Kane & Trochim, 2007). Taking this guidance, busy schedules and usual drop‐out rates into consideration, letters of invitation and participant information sheets were sent to 40 stakeholders, who met the eligibility criteria, at least a month before the first planned meeting. Consent forms were provided to participants on meeting days.

### 2.3 Data collection and data analysis

**Figure 1:**
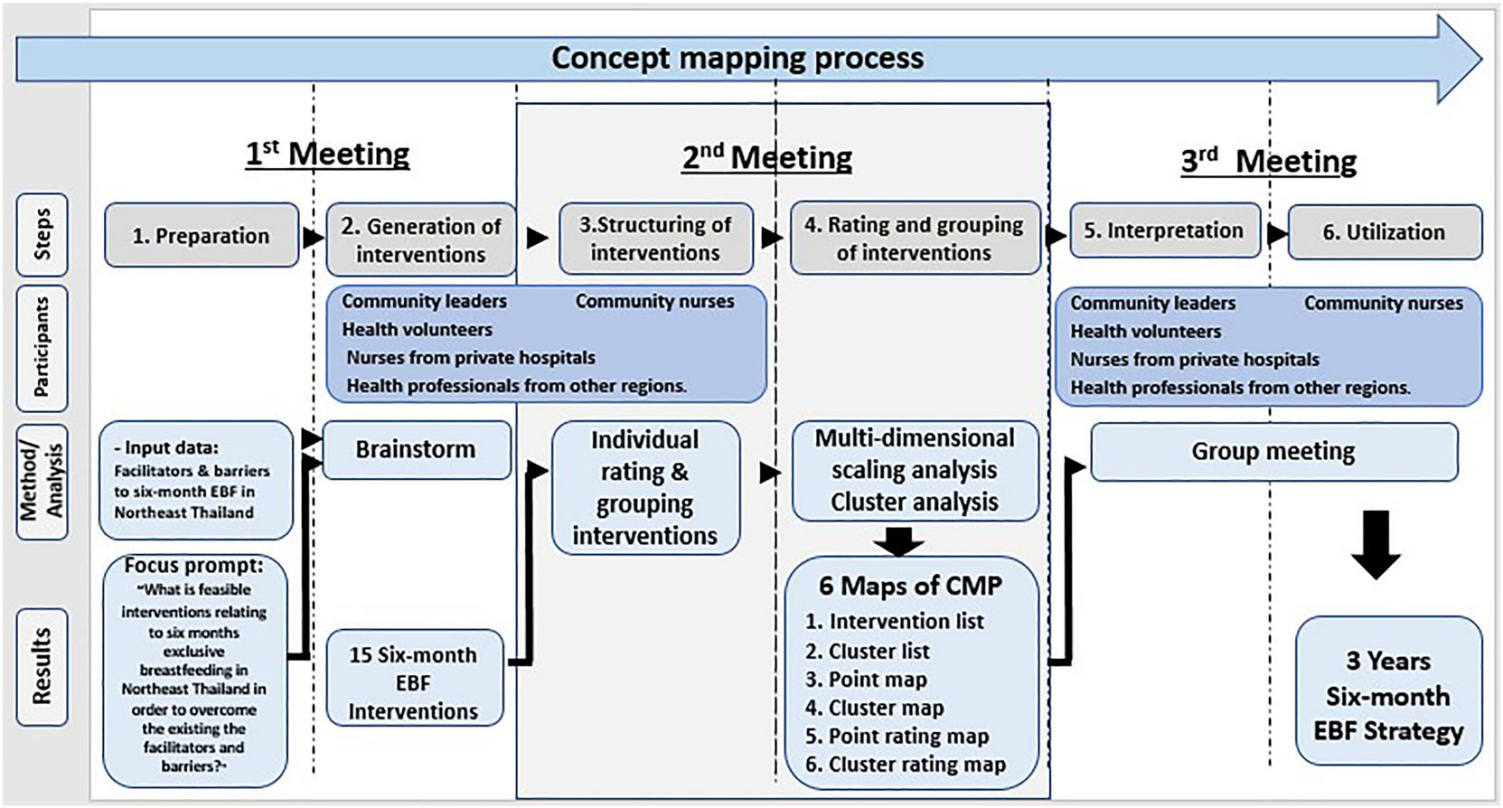
The overall concept mapping method including three meetings and six steps

The six CMP steps were incorporated into three meetings involving participants either as a group or individuals (Figure 1). All meetings were conducted in the Thai language.

During the first meeting, the participants were presented with an overview of relevant background information and findings regarding identifying and prioritising facilitators and barriers to 6‐month EBF in North‐east Thailand (Thepha et al., 2018; Thepha, Marais, Bell, & Muangpin, 2017). After the presentation, a brainstorming session was held in which participants were asked to suggest feasible intervention(s) to promote 6‐month EBF in North‐east Thailand. A feasible intervention was defined as an intervention that participants viewed as possible and implementable given the available time and resources. Participants were free to express any ideas regarding possible feasible interventions, which they wrote on “post‐it notes” and attached to a board visible to all participants. All proposed interventions were reviewed by two Thai‐speaking members of the research team (TT and SM). Duplicate and similar proposals were removed, and unclear ideas were clarified with the participants. The proposals were translated into English and discussed by four researchers (including native speakers of Thai and English) to ensure a common understanding of proposed interventions. Findings from this stage were then used as input data in the next stage.

During the second meeting, stakeholders were asked to individually rate the list of proposed feasible interventions from 1 (*low feasibility of intervention*) to 5 (*high feasibility of intervention*). They were also asked to categorise the interventions into groups based on the similarities of interventions in a way that made sense to them, for example, interventions related to family were grouped together. There was no limitation on the number of groups, and each grouping was assigned a unique name by the participants. The combination of multidimensional scaling analysis and cluster analysis was applied using SPSS version 24. In this process, the data were coded into a rectangular data matrix, in which each row or line represented an individual participant, and each column detailed an intervention (Kane & Trochim, 2007). This multidimensional scaling was used to locate each intervention as a separate point on a two‐dimensional (X, Y) map (Kane & Trochim, 2007). This analysis presented the relationship of each intervention on a visual graph called the point map. The average rating score for feasibility was added to the point map to develop a new point rating map (Kane & Trochim, 2007). The number of groups (clusters) was selected to attain the highest value of 6‐month EBF intervention model. Kane and Trochim (2007) have previously argued that there is no formula to get the “correct” number of clusters. The best number depends on the number of clusters, which provide the highest practical or value for solution or stakeholder or model Kane and Trochim (2007). In this study, the research team followed that approach and reviewed the data considering the characteristics and the relations between interventions. For example, Intervention 11 is close in distance to Cluster 1, but it is conceptionally and more similar to Intervention 5, and therefore, it was placed in Cluster 2. All interventions were first arranged in groups via dendogram numbers in order to identify the lower and upper boundaries for the number of appropriate groups. Second, the research team considered the characteristics of the intervention until that provided the highest value to the 6‐month EBF model. The cluster map was created in this step. The data analysis including all lists and maps, derived from multidimensional scaling analysis or cluster analysis, was confirmed as correct by two statisticians. The concept maps were used as input data in preparation for the third meeting.

The third meeting involved interpretation and utilisation processes of CMP. To interpret the results, the concept maps were visually presented to the participants. Initially, the researcher allotted time for participants to discuss, understand, and approve each map (approximately 10–15 min per map; Kane & Trochim, 2007), including assigning a final name to each cluster. The next step was to ask a probing question aimed at generating a plan for a 6‐month EBF intervention model “How would you develop a plan to increase six‐months EBF in North‐east Thailand?” The researcher provided sufficient time (approximately 45 min) for participants for discussion. The participants then prioritised the interventions based on their perceived importance, time, and resource requirements, which may include collaborations (Society for implementation science in nutrition (SISN, 2017). The final outcome was an intervention model, which included an implementation plan aiming to improve the 6‐month EBF rate in North‐east Thailand.

## 3 RESULTS

### 3.1 Participants

Forty participants were invited to take part in each of the three stages. Only 22, 21, and 17 participants took part in the first, second, and third meetings, respectively. Seventeen people attended all three meetings, four people attended two meetings, and one person attended one meeting. Participants included health care volunteers, health care professionals, community leaders, private hospital nurses, and health professionals from other regions (Table 1).

**Table 1:** Number of participants contributing to each concept mapping phase

Participants	Invited to each meeting	First meeting (group)	Second meetings (individual)	Third meeting (group)
Community leaders	7	5	2	1	
Health professionals at sub‐district level/community nurses	7	4	3	2	
Health volunteers/family members	20	11	15	12	
Private hospital nurses	4	1	1	—	
Health professionals from other regions	2	1	—	2	
TOTAL	40	22	21	17	

### 3.2 First meeting: Preparing and generating interventions

Eighty‐seven intervention ideas, including duplicate proposals, were proposed during the brainstorming session. There were various duplications. For example, 17 interventions, which were created from different stakeholders, regarding education for mother, were very similar in meaning and were combined to one intervention. Duplicate proposals were then removed to yield a total of 15 feasible interventions to be discussed in the next meeting (Table 2).

**Table 2:** The average feasibility score (out of 5) of the 15 proposed consensus interventions

Number	Proposed 6‐month EBF interventions	Average score
1	Antenatal education package for mothers	4.6
2	Education package for mothers who are working/working in another province	4.4
3	Education package for husbands	3.9
4	Education package for grandmothers, grandfathers, and nannies	4.3
5	Support groups for mothers to empower and encourage 6‐month EBF	4.4
6	Day‐stay service to provide antenatal/postpartum supportive environment for mothers in clinics	4.4
7	Monthly community meetings among health care professionals and head of communities to update or set up the policy for 6‐month EBF and share experiences of 6‐month EBF	4.1
8	School education package for teachers	4.0
9	Workplace education package for employers	4.2
10	Enforcement of the international code for the marketing of breastmilk substitutes (CODE)	4.0
11	Maternity professionals' training/workshop	4.6
12	Health care volunteer policy to support EBF	4.6
13	Maternity leave policy—lobbying government to increase maternity leave allowance for working mothers	4.2
14	Information transfer system policy/guidelines to improve postpartum mother information available between levels of health care	4.5
15	Health promotion activities such as an annual Ms breastmilk competition and healthy baby events	4.3

Abbreviation: EBF, exclusive breastfeeding.

### 3.3 Second meeting: Individualised structuring, rating, and grouping interventions

During this meeting, the 15 interventions were rated and grouped by individual participants. These data were analysed to calculate the average rating for each intervention (Table 2). The highest average score (4.6) was allocated for Intervention 1 “ante‐natal education package for mothers,” Intervention 11 “Maternity professionals' training/workshop,” and Intervention 12 “healthcare volunteer policy to support EBF.” The lowest average score was received by Intervention 3 “education package for husbands.”

**Figure 2:**
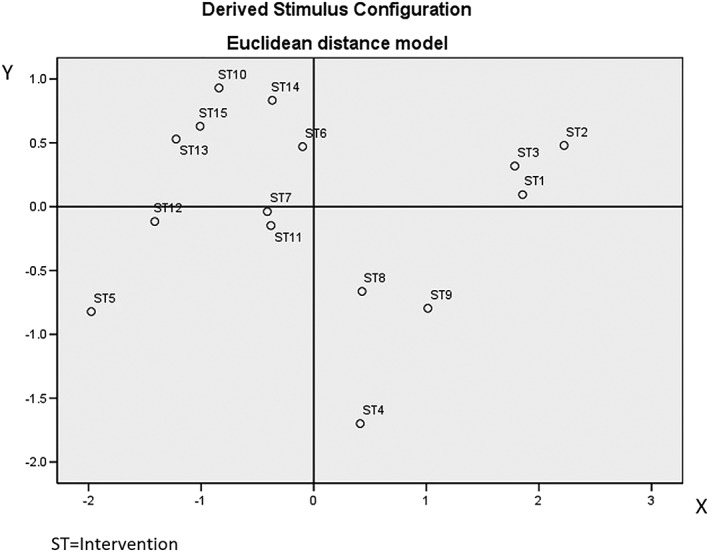
Point map showing the similarity between the 15 interventions

**Figure 3:**
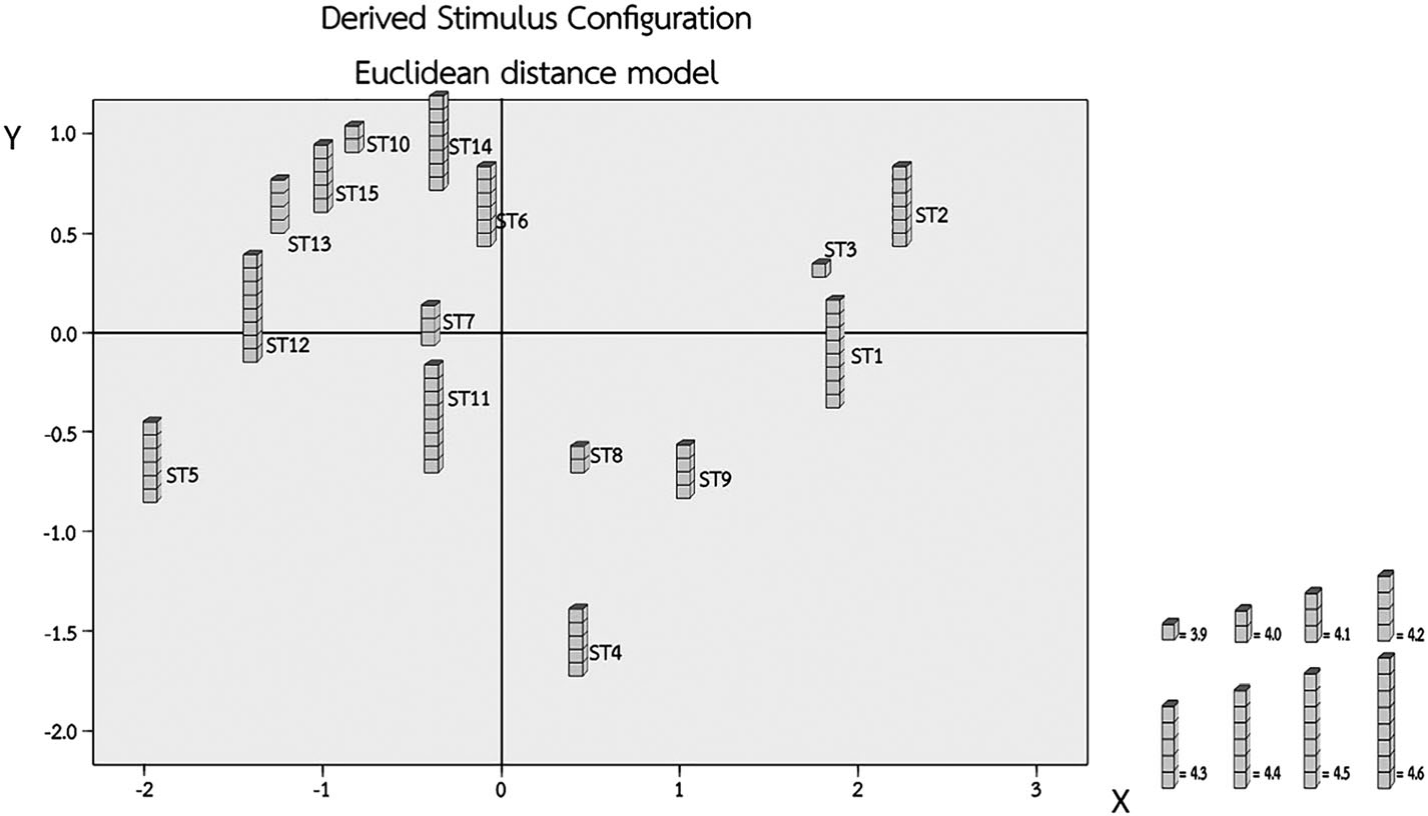
The point rating map showing the similarity between the 15 interventions as well as their feasibility

Further analysis was conducted using multidimensional scaling analysis to develop a point map, which is a visual‐data presentation (Figure 2). In the point map, each dot represents an intervention, and the distance between the dots represents the similarity of the interventions where the closest dots represent the most similar interventions. The average rating score of each intervention was added to the point map to develop the point rating map (Figure 3).

**Figure 4:**
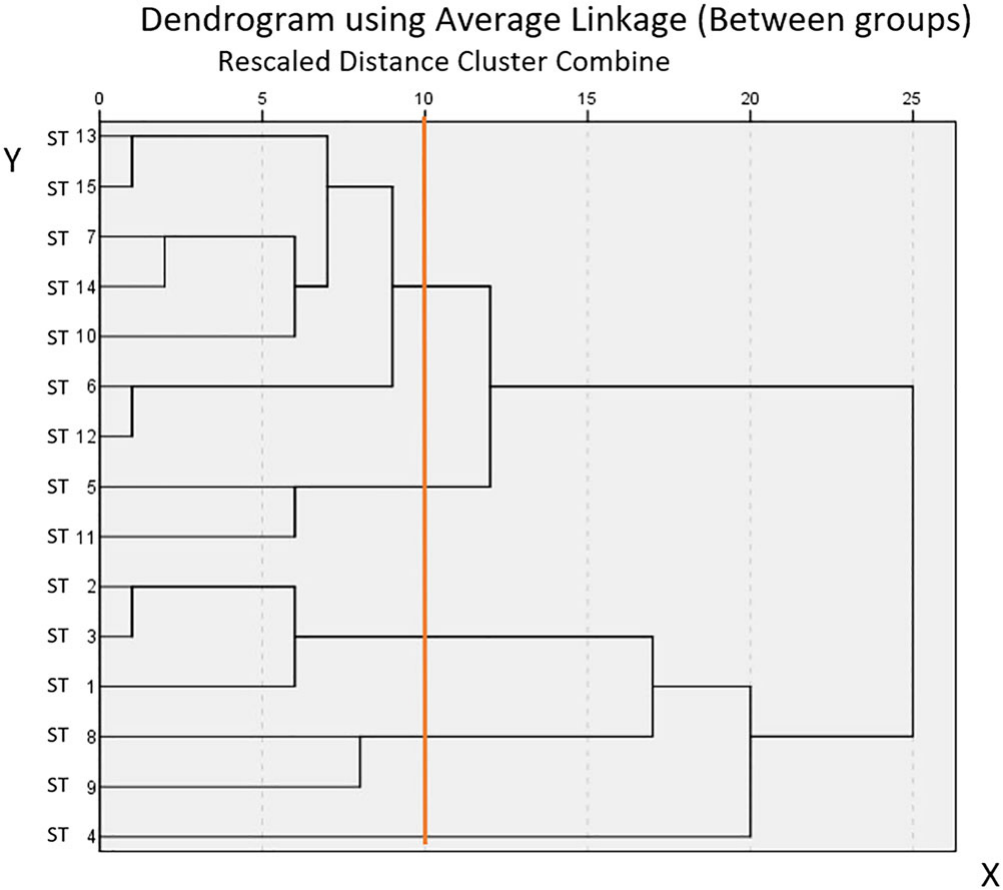
The dendogram graph shows the result of hierarchical cluster analysis with five groups (cluster) of 15 interventions

The next step involved identifying groupings of the interventions via hierarchical cluster analysis. To identify the number of clusters, the research team reviewed the analysed data, and consensus was reached at five clusters (Figure 4). Five groups (clusters) of interventions were identified: community services (Interventions 6, 7, 10, and 12–15), health care services (Interventions 5 and 11), education packages for parents (Interventions 1, 2, and 3), education packages for communities (Interventions 8 and 9), and education packages for families (Intervention 4; Table 3). The health care services group had the highest average score (4.5), whereas the education package for community groups had the lowest average score (4.1).

**Table 3:** The cluster lists of the feasible interventions and average score of each group

Six‐month EBF interventions	Average score
Cluster 1: Community services	4.3
Intervention 6: Day‐stay service to provide antenatal/postpartum supportive environment for mothers in clinics	
Intervention 7: Monthly community meetings among health care professional and head of community in order to update or set up the policy of 6‐month EBF, share the experience of 6‐month EBF	
Intervention 10: The enforcement of the international code of marketing of breastmilk substitutes (CODE)	
Intervention 12: Health care volunteer policy to support EBF	
Intervention 13: Maternity leave policy lobbying to government to increase maternity leave allowance for working mothers	
Intervention 14: Information transfer system policy/guidelines to improve postpartum mother information available between levels of healthcare	
Intervention 15: Annual Ms breastmilk or healthy baby (who is fed with breast milk) events	
Cluster 2: Health care services	4.5
Intervention 5: Support groups for mothers to empower and encourage 6‐month EBF	
Intervention 11: Maternity professionals' training/workshop	
Cluster 3: Education packages for parent	4.3
Intervention 1: Antenatal education package for mothers	
Intervention 2: Education package for mothers who are working/working in another province	
Intervention 3: Education package for husbands	
Cluster 4: Education packages for community	4.1
Intervention 8: School education package for teachers	
Intervention 9: Workplace education package to manager	
Cluster 5: Education package for family	4.3
Intervention 4: Education package for grandmothers, grandfathers, and nannies	

Abbreviation: EBF, exclusive breastfeeding.

**Figure 5:**
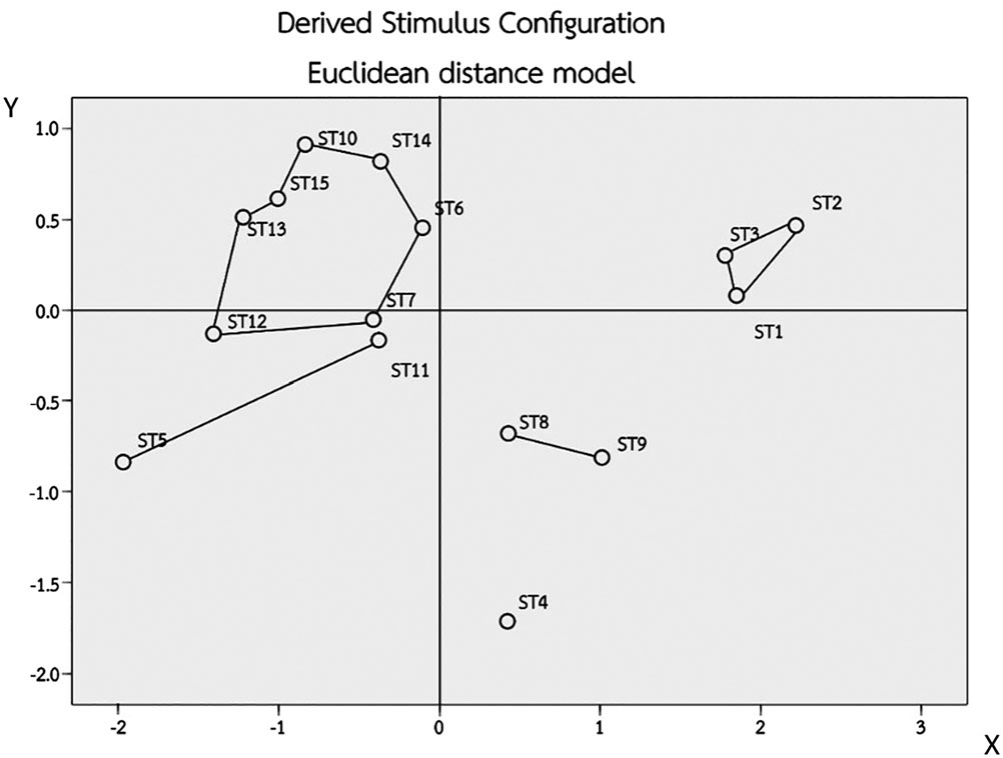
The cluster map showing the relationship between the 15 interventions

**Figure 6:**
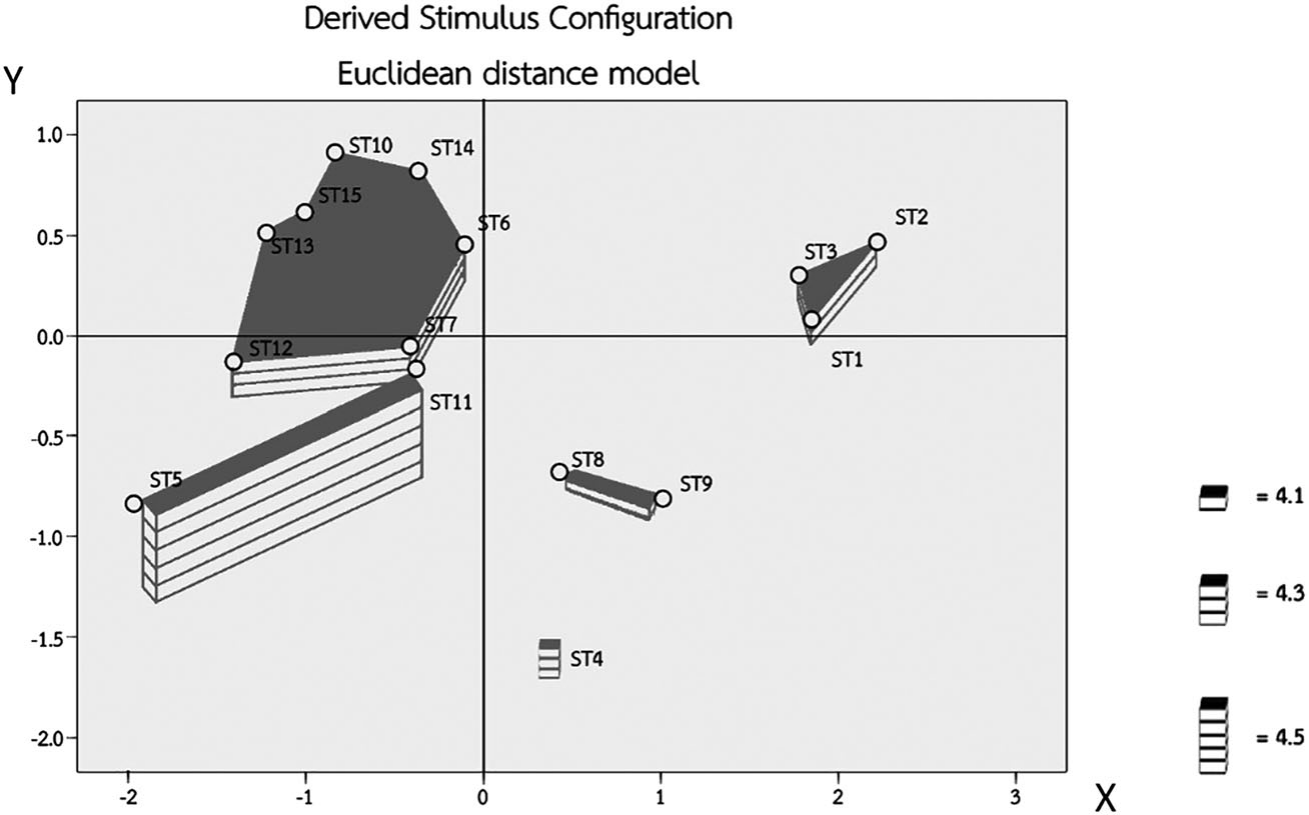
The cluster rating map showing the relationship between the 15 interventions and their feasibility

Finally, a cluster map was created via hierarchical cluster analysis (Figure 6). This map illustrated how the interventions were grouped (Figure 5). The average rating score of each group was added to the cluster map to produce a cluster rating map (Figure 6).

### 3.4 Third meeting: Interpretation and utilisation of an intervention strategy

**Figure 7:**
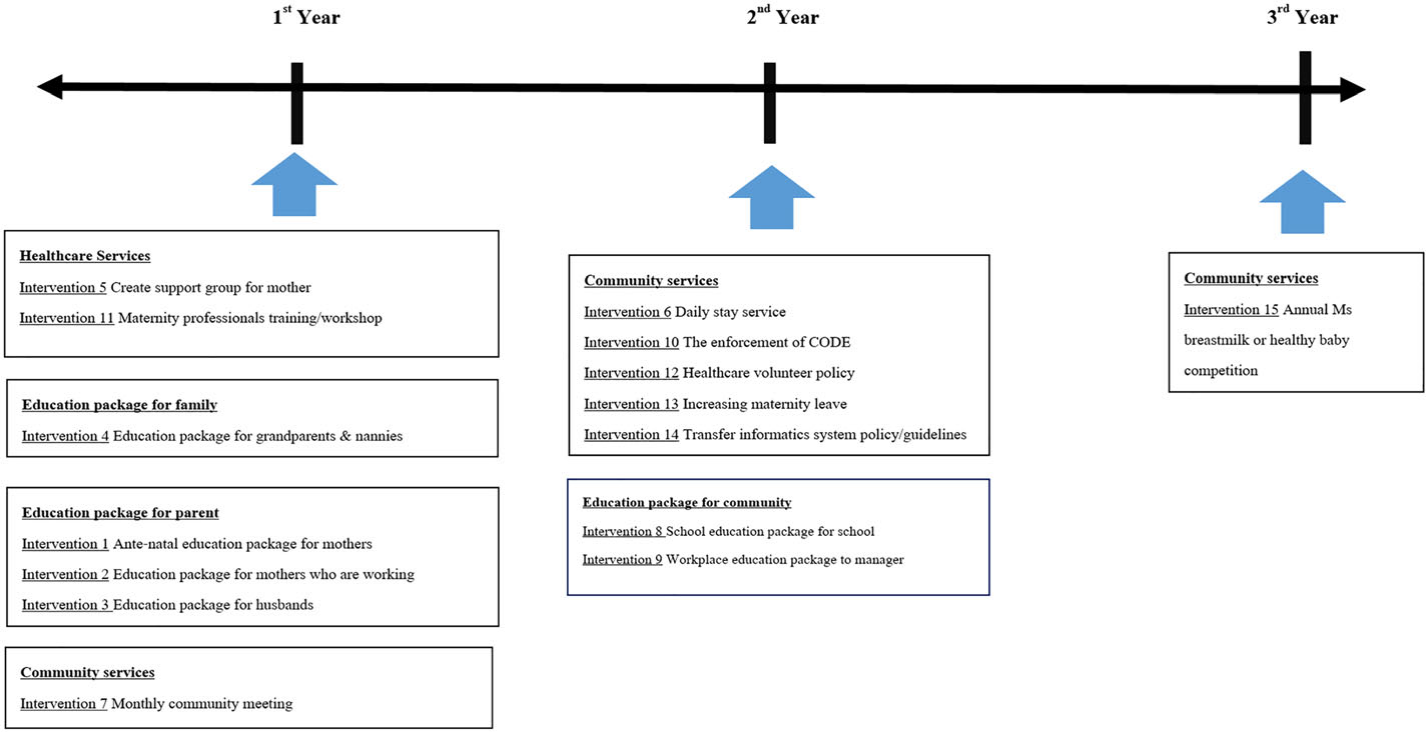
A 3‐year 6‐month exclusive breastfeeding intervention strategy model for North‐east Thailand

Drawing on the previous findings, stakeholders developed an intervention strategy, which included all five clusters. The duration required to implement all 15 interventions was agreed to be 3 years (Figure 7). The health care services cluster, education packages for families, and the education packages for parents were suggested as interventions for the first year. The health care services cluster was chosen as a top priority, and importantly, “intervention 11: maternity professionals' training/workshop” was prioritised as the first intervention for the pilot study. Most interventions in the community services and education packages for community clusters were selected for implementation in the second year. Lastly, health promotion opportunities were proposed for the third year. All results were approved by the stakeholders who expressed their willingness to incorporate them into health care policy in their communities.

## 4 DISCUSSION

A 3‐year intervention strategy model was developed following an implementation research approach for the development of a complex intervention. Fifteen interventions were proposed based on previously identified specific facilitators and barriers in the North‐east Thailand (Thepha et al., 2017; Thepha et al., 2018). Notably, several interventions included in this model have been implemented successfully in other countries. Some examples include interventions for training health care professionals through sharing knowledge (Haroon, Das, Salam, Imdad, & Bhutta, 2013; Kupratakul et al., 2010), training health care and nutrition workers to promote EBF in communities (Bhandari et al., 2003), web‐based interventions (Edwards et al., 2015; Kahin et al., 2017; Sigman‐Grant & Kim, 2016), community‐based interventions in Pakistan and the United Kingdom (Bhutta et al., 2008; McInnes, Love, & Stone, 2000), home visits and breastfeeding support (Coutinho, Lira, Lima, & Ashworth, 2005), and education packages for parents in Taiwan, Turkey, and Jordan (Aksu, Küçük, & Düzgün, 2011; Huang et al., 2007; Khresheh, Suhaimat, Jalamdeh, & Barclay, 2011; Lin, Chien, Tai, & Lee, 2008). What makes our model more likely to succeed than previous programmes is the combination of different interventions, including training, support, health promotion and individual, family, and social interventions. Indeed, combinations of interventions have been shown to be more successful than individual interventions (Kim, Park, Oh, Kim, & Ahn, 2018). Another consideration for our model being successful is basing it on experiential knowledge instead of the top‐down approach, which is usually followed by relying on “expert opinion” (Apasakul, 2016; National Statistical Office Thailand, 2013). The proposal by the stakeholders to implement the intervention across 3 years is in line with most strategic plans in the health sector, which are 3 to 5 years in duration (NACCHO 2012). To ensure sustainability, it is recommended that the breastfeeding gear model is followed to guide in the scaling up of this intervention strategy (Perez‐Escamilla, Curry, Minhas, Taylor, & Bradley, 2012).

### 4.1 Strengths and limitations

CMP was beneficial for both researchers and participants. It helped the researcher capture different dimensions through a combination of qualitative and quantitative methods. In this study, the six steps of CMP were systematically conducted to attain stakeholder consensus regarding intervention feasibility that was both strategic and tailored to the local context. For example, in the preparation step, a wide range of stakeholders was included to provide diverse perspectives and ideas, contributing to realistic interventions appropriate for North‐east Thailand. These results were systematically analysed and presented in the interpretation and utilisation steps using visual data to help the stakeholders easily understand and interpret the data. In a similar way, stakeholders were encouraged to express their ideas through pictures rather than research language, and this provided useful information about the feasibility of each interventions and the relationships between interventions. Furthermore, the three meetings allowed stakeholders to express their ideas both individually and in groups via brainstorming, rating, grouping, and group discussions.

Considering limitations, our study results, although highly relevant in the local setting, may not be directly applicable to other areas. The main challenge remaining is to incorporate the intervention model into the health care policy of the region and to consider its suitability for Thailand as a whole. Other limitations pertain to the time commitment, identification of potential facilitators to run the whole CMP as well as stakeholders. The ratio of participants among health care professionals, community leaders, and health care professionals from other regions was not calculated. This may affect to the results especially rating, grouping, or prioritisation of interventions.

## 5 CONCLUSION

A 3‐year intervention strategy model including 15 interventions was identified as a complex intervention model specific to North‐east Thailand. This model was developed based on local data and knowledge, using a systematic CMP process involving stakeholder consensus on proposed interventions. CMP could be applied to address other areas and issues. In the future, this model could be implemented and incorporated into the health policy in North‐east Thailand. The effectiveness of each intervention and the overall model need to be evaluated and reviewed accordingly to attain a sustainable model prior to scaling it up to other areas. If the model is proved to be sustainable, it could be supported by the government.

## CONFLICTS OF INTEREST

The authors declare that they have no conflicts of interest.

## CONTRIBUTIONS

All authors made substantial contributions to the conception and design of the research. TT conducted the concept mapping process, analysed the data, and drafted the article. DM, JB and SM provided critical feedback for data analysis and revision of the article. All authors gave final approval for the article.
